# Hepatic abscess – clinical aspects of *Klebsiella* infection – a case report

**DOI:** 10.1186/1471-2334-13-S1-P82

**Published:** 2013-12-16

**Authors:** Amelia Blescun

**Affiliations:** 1 “Dr. Teodor Andrei” Lugoj County Hospital, Lugoj, Romania

## Background

The reported incidence of hepatic abscess ranges from 1.1 to 3.6 per 100,000 cases, being considered one of the differential diagnoses for fever of unknown origin. Theoretically, *Klebsiella* is the second most frequent cause of pyogenic abscess (18%). During the past 6 months, 3 cases of hepatic abscess were diagnosed at the “Dr. Teodor Andrei” Lugoj County Hospital.

## Case report

A 61-year-old patient from Lugoj was admitted accusing fever, chills and dysuria for approximately 3 days. He was first diagnosed with diabetes mellitus, which required high insulin doses for correction. Clinical and laboratory findings demonstrated sepsis (fever, chills, leukocytosis and neutrophilia, high glycemic levels, renal failure). X-ray and echography were normal. Blood cultures were negative; however, urine culture was positive for *Klebsiella*. He was administered ceftriaxone and gentamicin i.v., according to the antibiotic susceptibility tests. Unfortunately, during admission, jaundice, high ALT, AST and cholestasis complicated the case. CT scan revealed a trans-sonic inhomogeneous image suggesting a hepatic abscess, therefore the patient was sent to immediate surgery, which revealed a pyogenic collection. Cultures were positive for *Klebsiella*. The postoperative evolution slowly improved only after local drainage and antibiotics.

## Conclusion

By presenting this case report, we tried to point out the necessity of further investigations for other causes of fever in patients who do not respond to initial therapy. Additionally, positive urinary cultures for *Klebsiella* may be interpreted as secondary to the initial bacteremia from the hepatic septic source, and initial diagnosis of urinary infection had to be reassessed. The evolution of the patient was favorable only after the surgical drainage of the abscess.

